# Hepatic microvascular dysfunction and increased advanced glycation end products are components of non-alcoholic fatty liver disease

**DOI:** 10.1371/journal.pone.0179654

**Published:** 2017-06-19

**Authors:** Evelyn Nunes Goulart da Silva Pereira, Raquel Rangel Silvares, Edgar Eduardo Ilaquita Flores, Karine Lino Rodrigues, Isalira Peroba Ramos, Igor José da Silva, Marcelo Pelajo Machado, Rosiane Aparecida Miranda, Carmen Cabanelas Pazos-Moura, Cassiano F. Gonçalves-de-Albuquerque, Hugo Caire de Castro Faria-Neto, Eduardo Tibiriça, Anissa Daliry

**Affiliations:** 1 Laboratory of Cardiovascular Investigation, Oswaldo Cruz Institute, Rio de Janeiro, RJ, Brazil; 2 Laboratory of Celular and Molecular Cardiology, Federal University of Rio de Janeiro, Rio de Janeiro, RJ, Brazil; 3 National Center of Structural Biology and Bio-imaging, Federal University of Rio de Janeiro, Rio de Janeiro, RJ, Brazil; 4 Laboratory of Pathology, Oswaldo Cruz Institute, Rio de Janeiro, RJ, Brazil; 5 Laboratory of Molecular Endocrinology, Federal University of Rio de Janeiro, Rio de Janeiro, RJ, Brazil; 6 Laboratory of Immunopharmacology, Oswaldo Cruz Institute, Rio de Janeiro, RJ, Brazil; IDIBAPS Biomedical Research Institute, SPAIN

## Abstract

**Background:**

This study aimed to investigate the pathophysiology of hepatic microcirculatory dysfunction in non-alcoholic fatty liver disease (NAFLD).

**Methods:**

In Wistar rats, NAFLD model was induced by 20 weeks of high-fat diet (HFD) feeding. Rolling and adhesion of leukocytes and tissue perfusion in hepatic microcirculation were examined using in vivo microscopic and laser speckle contrast imaging (LSCI), respectively. Oxidative stress and inflamatory parameters were analysed by TBARs, catalase enzyme activity, RT-PCR and ELISA. The participation of advanced glycation end-products (AGE) and its receptor RAGE was evaluated by the measurement of gene and protein expression of RAGE by RT-PCR and Western-blot, respectively and by liver and serum quantification of fluorescent AGEs.

**Results:**

Wistar rats fed high-fat diet (HFD) showed increase in epididymal and abdominal fat content, systolic arterial blood pressure, fasting blood glucose levels, hepatic triglycerides and cholesterol, and impairment of glucose and insulin metabolisms. Liver histology confirmed the presence of steatosis and ultrasound analysis revealed increased liver size and parenchymal echogenicity in HFD-fed rats. HFD causes significant increases in leukocyte rolling and adhesion on hepatic microcirculation and decrease in liver microvascular blood flow. Liver tissue presented increase in oxidative stress and inflammtion. At 20 weeks, there was a significantly increase in AGE content in the liver and serum of HFD-fed rats and an increase in RAGE gene expression in the liver.

**Conclusion:**

The increase in liver AGE levels and microcirculatory disturbances could play a role in the pathogenesis of liver injury and are key components of NAFLD.

## Introduction

Excess of caloric intake characteristic of the western diets is linked to a significant increase in metabolic syndrome (MS) prevalence worldwide [1]. The MS is a cluster of dysfunctions including abdominal obesity, hypertriglyceridemia, hypertension, low high-density lipoprotein-cholesterol levels, and glucose intolerance or insulin resistance that identify patients at increased risk of type 2 diabetes (T2DM) and cardiovascular disease [2,3]. Liver abnormal fat accumulation is a feature of non-alcoholic fatty liver disease (NAFLD) and strongly associated with the MS [4].

NAFLD is one of the most common chronic liver diseases worldwide, characterized by increased hepatic fat accumulation in individuals in the absence of excessive alcohol consumption [5]. Epidemiological studies suggest that patients with NAFLD are at increased risk of developing T2DM [6]. NAFLD includes a wide liver disorder spectrum from simple steatosis to non-alcoholic steatohepatitis (NASH), in which steatosis progresses to hepatocellular injury, with lobular inflammation and fibrosis [7]. NAFLD increases the risk of end-stage liver disease, liver cirrhosis and hepatocellular carcinoma [8]. However, the factors that determine whether patients will progress from simple hepatic steatosis to NASH remain unclear. According to the “multiple hit” hypothesis several parallel factors synergistically contribute to disease progression, including insulin resistance and liver fat accumulation [9]. Several recent studies have suggested that advanced glycation end products (AGEs) could act as one of these factors and therefore play an important role in the pathogenesis of NAFLD [10,11].

AGEs are products of non-enzymatic reactions between reducing sugars and proteins, lipids or nucleic acids [12]. AGEs have their production increased in several pathological conditions, such as diabetes, renal failure, neurodegenerative diseases, NASH and cirrhosis [13,14,15]. In experimental models of NAFLD, AGEs have been described to exacerbate liver injury, fibrosis and inflammation [10,11,16] mainly through stimulation of the receptor for advanced glycation end products (RAGE) which in turn activate oxidative and inflammatory pathways [10,17].

Experimental models of NAFLD exhibit systemic endothelial dysfunction and increased cardiovascular risk also seen in NAFLD patients [18,19,20]. Interestingly, most complications leading to morbidity in MS are from vascular origin [21], suggesting that the vasculature is a key target in MS. Considering that AGE and RAGE are pivotal participants in vasculature injury in several diseases, including diabetes and Alzheimer [15,22], and that Kupffer cells and liver sinusoidal endothelial cells (LSEC) are the major cellular sites of AGE uptake and clearance [23,24,25], AGE accumulation in the liver could participate in hepatic microvascular damage, further exacerbating liver dysfunction and contributing to NAFLD progression. To date, few studies addressed the hepatic microcirculation status in NAFLD, especially in the early stages of the disease.

Therefore, in the present study, we tested the hypothesis that hepatic microvascular dysfunction is a feature of early stage of NAFLD induced by the intake of high-fat diet (HFD). Moreover, to further explore the potential mechanisms involved in NAFLD pathogenesis, we also studied the AGE-RAGE pathway, oxidative stress and markers of inflammatory response in the liver of HFD-fed rats.

## Materials and methods

### Animals and experimental protocol

Twenty male Wistar rats from the central animal facility of the Oswaldo Cruz Foundation, Brazil, were used in the study. The rats (4 weeks of age) were housed in standard cages in a temperature-controlled room (22 ± 1°C) with a 12-h light/dark cycle. The experimental model of NAFLD or MS was induced in 10 animals by 20 weeks of feeding with a high-fat diet administration as previously described by or group [26]. The high-fat diet consisted of a standard rat diet modified containing 30% fat, 56% carbohydrate, and 14% protein (% g) [26]. The main fat source of the high fat diet was saturated fat (lard). The non-MS control (CTL) animals received standard rat diet (n = 10) for 20 weeks. All tissues, samples and analyses were collected and carried out at the end of the experimental protocol. After intravital microscopy and laser speckle contrast imaging evaluations, animals under anaesthesia (ketamine 100 mg/kg and xylazine 10 mg/kg, via i.p.) were subjected to cardiac puncture for euthanasia and total blood collection. Liver and adipose tissue were collected. Visceral and epididymal adipose tissue deposits were dissected and weighed. Plasma was separated by centrifugation at 3000 rpm for 15 min at 4°C, and the plasma aliquots were stored at -80°C until measurement. All of the experimental procedures were conducted in accordance with the internationally accepted principles for the Care and Use of Laboratory Animals and were approved by the Oswaldo Cruz Foundation Animal Welfare Committee (CEUA license L-034/2016).

### Biochemical parameters

%HbA_1c_, hepatic cholesterol and triglycerides, total bilirubin, vitamin D, and the enzyme activitiy of alanine aminotransferase (ALT) were analysed using commercial kits with the Cobas c system (Roche Molecular Biochemicals, Indianapolis, IN). Fasting blood glucose levels were measured using an automatic glucose monitor (One Touch Ultra2, Johnson & Johnson Medical S.A., Buenos Aires Argentina).

### Blood pressure

Systolic blood pressure (SBP) was measured by non-invasive measurement of the tail pressure (BP-2000, Visitech Blood Pressure Analysis System, Visitech Systems Inc., Apex, NC, USA).

### Oral glucose tolerance test (OGTT) and intraperitoneal insulin tolerance test (IpITT)

Oral glucose tolerance test (OGTT) was performed in animals that had fasted overnight. Blood glucose levels were measured 0, 30, 60, 90, 120 and 150 min after the administration of a glucose overload (2 g/kg), and the areas under the glucose curves (AUCs) were calculated. Intraperitoneal insulin tolerance test (IpITT) were performed in 6 h fasted rats which were given an i.p. injection of insulin (1 U/kg), and blood glucose levels were measured at 0, 15, 30, 60, 90, 120, and 150 minutes after injection. An automatic glucose monitor was used to the measure blood glucose levels in the venous blood (One Touch Ultra2, Johnson & Johnson Medical S.A., Buenos Aires Argentina).

### Western blot

Western blotting were used to study the protein expression of insulin receptor (IR-β), phosphorylated IR-β (*p*-IRβ), adenosine monophosphate-activated protein kinase (AMPK), phosphorylated adenosine monophosphate-activated protein kinase (*p*-AMPK), phosphorylated insulin receptor substrate 1 (*p*-IRS1), protein-tyrosine phosphatase 1B (PTP1B) and receptor for AGE (RAGE). All the primary antibodies (IR-β, *p*-IRβ, AMPKα1/2, *p*-AMPKα1/2, *p*-IRS1, PTP1B and RAGE) were purchased from Santa Cruz Biotechnology (Santa Cruz, CA, USA) and the dilution was 1:1000, with the exception of RAGE, which was 1:500. Briefly, liver tissue (left lobule) was homogenized in lysis buffer (20 mM Trizma, 137 mM NaCl, 10% Glycerol, 1% Nonidet P-40 and 2 mM EDTA) containing a protease inhibitor cocktail (Roche Diagnostics, IN, USA) and centrifuged for 45 min at 14 000 g, 4°C. After centrifugation, the total protein content of the supernatant was quantified using a Pierce^™^ BCA Protein assay kit (Thermo Scientific, MA, USA). Then, 15–30 μg of protein per lane was separated on a 12% SDS gel and transferred to a nitrocellulose membrane (Bio-Rad Laboratories, Munich, Germany). After blocking with 5% BSA (Sigma-Aldrich, Germany), the membranes were incubated overnight at 4°C with primary antibodies. For RAGE detection, a secondary RDye 680RD Donkey-anti-Goat Antibody (1:15000; LI-COR Biosciences, Cambridge, UK) was used. The bound complex was detected using the Odyssey Infrared Imaging System (Li-Cor; Lincoln, NE, USA). The images were analysed using the Image Studio Lite Software version 4.0.21 (Li-Cor) to obtain the integrated intensities and normalized with respect to the GAPDH signals (mouse monoclonal anti-GAPDH antibody, 1:30000; Fitzgerald Industries International, USA, followed by a secondary IRDye 800CW Goat-anti-Mouse Antibody (1:20000; LI-COR Biosciences, Cambridge, UK). For IR-β, *p*-IRβ, AMPKα1/2, *p*-AMPKα1/2, *p*-IRS1 and PTP1B detection membranes were incubated with peroxidase-labeled anti-rabbit IgG antibody (Amersham Biosciences, Inc.; 1:10,000 dilution), antimouse IgG antibody (Cell signaling; 1:10,000) or antigoat IgG antibody (Amersham Biosciences, Inc.; 1:20,000 dilution) for 2h at room temperature. The blots were washed and incubated with a luminogen detection reagent (Amersham ECL Prime Western Blotting Detection reagent; Amersham Biosciences, BKM, England). Chemiluminescent signal was detected by ImageQuant LAS 4000 equipment and followed by densitometric analyses (GE Healthcare Life Sciences, BKM, England). Cyclophilin (Thermo Scientific, MA, USA) was used for loading control.

### Histopathology

Livers were fixed in Millonig´s phosphate buffered formalin solution at pH 7.2, embedded in paraffin, and processed for light microscopy (hematoxylin and eosin (HE) staining). Steatosis was analysed by the presence of micro and macrovesicles in the HE-stained sections. The infiltration of polymorphonuclear cells was assessed in HE staining sections, based on the localization of the cell and morphology of the nucleus of the cell. For HE staining five histological sections from ten animals per group were analysed. Oil-red staining was performed in frozen tissue according to previously described methodology [27]. Tissue lipid accumulation was quantified in four histological sections from three animals per group via the amount of ORO staining by using standard quantification software ImajeJ [27,28]. The severity of steatosis was determined by the percentage of steatotic hepatocytes: <33% (mild), 33–66% (moderate) and >66% (severe)[29].

### Ultrasound analyses

To perform the liver ultrasound analyses, the animals were anesthetized with isoflurane 2%. After abdominal hair was shaved, rats were positioned supine on a heated table. A sound conductive gel (Carbogel®, Brazil) was applied to the animal and the ultrasound examination was performed with the aid of the VEVO 770 system (VisualSonics, Canada) coupled to a 30 MHz transducer and the MyLabTM30 cardiovascular system (Esaote, Italy) coupled to a transducer 7.5–10 Mhz. The aspect and echogenicity of the hepatic parenchyma, their relationship with the renal cortex, the portal vein diameter, area and transverse diameter of the liver were analysed. The same experienced technical observer performed all tests without knowing the experimental groups to which they belonged.

### Intravital microscopy

For intravital microscopy of the liver [30,31], overnight-fastened animals were anesthetized via intraperitoneal (i.p.) injection with ketamine (100 mg/kg) and xylazine (10 mg/kg). A midline and a left subcostal incision were made to exteriorize the liver. The hepatic ligaments were dissected and the intestine was covered with a saline-soaked gauze to minimize tissue dehydration. The left liver lobe was then exteriorized and placed on a glass disk and covered with a glass slide for microcirculation analyses. With the use of a 10X ocular and 10X objective (Olympus BX150WI; Center Valley, PA, USA) images were displayed on a television monitor and recorded by a digital video recorder (DP73; Olympus, MA, USA) for off-line analysis with the Cellsens standard 1.9 software program (Olympus, MA, USA). The leukocyte–endothelial interaction was evaluated by counting the number of labelled leukocytes (0.3 mg/kg rhodamine 6G, i.v.) rolling or adhering to sinusoids and post-sinusoidal venules. Rolling leukocytes were defined as white blood cells with a slower velocity than the erythrocytes and a detectable rolling motion. Leukocytes that remained stationary on the sinusoidal or venular endothelium for 30 s or longer were considered adherent cells. Rolling and adherent cells were counted in a 170 μm^2^ area comprising sinusoids and post-sinusoidal venules.

### Laser speckle contrast imaging

Hepatic microvascular blood flow was measured by Laser Speckle Contrast Imaging apparatus (Pericam PSI system, Perimed, Sweden), which is a method that provides a microcirculatory perfusion index proportional to the concentration and mean velocity of red blood cells used to assess microvascular blood flow in real time [32,33]. The surgical procedure was performed as for intravital microscopy, as described above. The animals were maintained on a stable surface, in a room with a constant temperature at 25°C and placed under a laser light system with image contrast with a wavelength of 785 nm for continuous measurement of tissue blood perfusion in real time. The distance between the scan head and the liver surface was approximately 10 cm. Relative liver blood flow of all animal groups was expressed as arbitrary perfusion units (APUs).

### Thiobarbituric acid reactive species (TBARs)

Lipid peroxidation in the liver was assessed by measuring malondialdehyde (MDA) concentrations using thiobarbituric acid [34]. The livers were homogenized in cold phosphate buffer, pH 7.4 with BHT (final concentration 0.2%). The samples (0.5 mL) were mixed with an equal volume of thiobarbituric acid 0.67% (Sigma Chemical Co., St. Louis, MO) and then heated at 96°C for 30 min. TBARs were determined by the absorbance at 535 nm. The results are expressed as malondialdehyde levels (MDA, ε = 1.56 × 105 M− 1 cm− 1)(Draper et al., 1990).

### Catalase activity

Livers were collected and homogenized in KPE buffer (0.1 M potassium phosphate buffer with 5 mM EDTA disodium salt, pH 7.5) in the proportion of 1:9 w/v liver/buffer. The samples were centrifuged at 600 g for 10 min, at 4°C, and the supernatant was used in 1:10 dilution for the estimation of catalase activity. For this purpose, the enzymatic decomposition of hydrogen peroxide was followed continuously at 240 nm. For this, 1 μL of samples was added to a microplate, and 97 μL of hydrogen peroxide 0.16% in PBS (pH 7.0) was used as the substrate. Enzymatic activity was expressed as mmol hydrogen peroxide decomposed/min/mg protein, according to the method described by Aebi [35].

### TNF-α and IL-1β measurements

Measurements of TNF-α and IL-1β were done on serum and liver samples using commercially available ELISA kit (R&D Systems, Minneapolis, USA), in accordance with the manufacturer’s instructions.

### RT-PCR

Total RNA was isolated from the liver (aliquots from the left lobe) using the RNeasy Mini Kit (Qiagen). cDNA was synthesized with a high capacity cDNA reverse transcription Kit (Applied Biosystems) from 1 μg of total RNA in a final volume of 20 μl. The primers used for PCR amplification were as follows: NADPH oxidase p47 subunit (N47), forward 5′-GTGAAGCCATCGAGGTCATTC-3′ and reverse 5′-CCCGCGGCTTCTAATCTGT-3′; eNOS, forward 5´-GTATTTGATGCTCGGGACTG-3´ and reverse 5´-AGATTGCCTCGGTTTGTTG-3´;

IL-6 forward 5´-AATCTGCTCTGGTCTTCTTGGAG-3´ and reverse 5´-GTTGGATGGTCTTGGTCCTTAG-3´; VCAM, forward 5´-GCGAAGGAAACTGGAGAAGACA-3´ and reverse 5´-ACACATTAGGGACCGTGCAGTT-3´; Catalase (CAT), forward 5′- ACTCAGGTGCGGACATTC-3′ and reverse 5′- GGAGTTGTACTGGTCCAGAAGAGCC -3′; RAGE (RAGE), forward 5′- CAGGGTCACAGAAACCGG -3′ and reverse 5′- ATTCAGCTCTGCACGTTCCT -3′ and β-actin (β-a), forward 5′- CCACCCGCGAGTACAACCTTCTT -3′ and reverse 5′- GAAGCCGGCCTTGCACATGCC -3′. Real-time PCR was performed with the power SYBR Green PCR Master Mix (Applied Biosystems) according to the manufacturer's instructions. All of the PCR amplifications were carried out using a 7500 real time PCR system (Applied Biosystems). The expression of target genes was normalized to the expression of β-actin, and the ΔΔ^Ct^ method was used for gene expression determination.

### Quantification of advanced glycation end products (AGE)

Liver and serum concentrations of fluorescent AGEs were determined by the method of Nakayama [36]. Briefly, the fluorescence of AGEs samples was measured at an emission wavelength of 440 nm and an excitation wavelength of 370 nm against a blank of 0.1 N NaOH solution on a SpectraMax M5 ELISA Microplate Reader (Molecular Devices, CA, USA)(DCCT, 1993). A native BSA preparation (1 mg/ml of 0.1 N NaOH) was used as a reference, and its fluorescent intensity was defined as one unit of fluorescence. The fluorescence values of the samples were measured at a protein concentration of 1 mg/ml and are expressed in AU compared with the native BSA preparation.

### Statistical analysis

Data are expressed as the mean ± SEM for each group. Student’s *t* test was used for statistical significance analysis between the two groups. Differences with *P* values of less than 0.05 are considered significant.

## Results

### High-fat diet-induced metabolic alterations

Wistar rats were fed standard chow diet (CTL) or high-fat diet for 20 weeks (HFD). At the end of treatment, HFD rats displayed the same weight than CTL (Table 1). However, high-fat diet induced a 144% and 91% increase in epididymal and abdominal fat content, respectively (Table 1). We did not observe renal damage in the HFD-fed rats, as assessed by 24h urine collection and analyses of micro or macroalbuminuria, and serum urea and creatinine levels (data not shown). %HbA_1c_ and total bilirubin were not altered after 20 weeks on HFD while vitamin D was significantly decreased in the HFD-fed rats (Table 1). ALT serum level in HFD-fed rats was not altered compared with controls (Table 1). Rats fed the HFD had higher hepatic triglycerides and cholesterol levels compared to those fed the control diet (Table 1). After 20 weeks on HFD, rats exhibited an increase in systolic arterial blood pressure (Table 1).

**Table 1:** Hemodydamic and metabolic parameters in rats fed control or high-fat diet (HFD) for 20 weeks.

**Parameters**	**Control (n = 10)**	**HFD (n = 10)**
Weight (g)	447.8 ± 12	468.5 ± 20
Epydidimal fat content (g)	6.7 ± 0.4	16.36 ± 1.2***
Abdominal fat content (g)	4.2 ± 0.3	8.03 ± 0.9***
Systolic arterial blood pressure (mmHg)	138.8 ± 1.8	146.3 ± 4.5*
Fasting blood glucose (mmoL/L)	4.4 ± 0.2	5.6 ± 0.2**
HbA1c (%)	3.8 ± 0.04	3.9 ± 0.02
Total bilirubin (mg/dL)	0.06 ± 0.009	0.08 ± 0.005
25-hydroxy Vitamin D (ng/mL)	59.9 ± 2.1	39.1 ± 7.7*
ALT (U/L)	67.5 ± 9.2	67.0 ± 16.1
Liver triglycerides (mg/dL)	81.1 ± 7.0	233.6 ± 26.2***
Liver cholesterol (mg/dL)	15.3 ± 0.2	26.7 ± 2.5***

*P < .05 versus control;

**P < .01 versus control ;

***P < .001 versus control.

**Fig 1:**
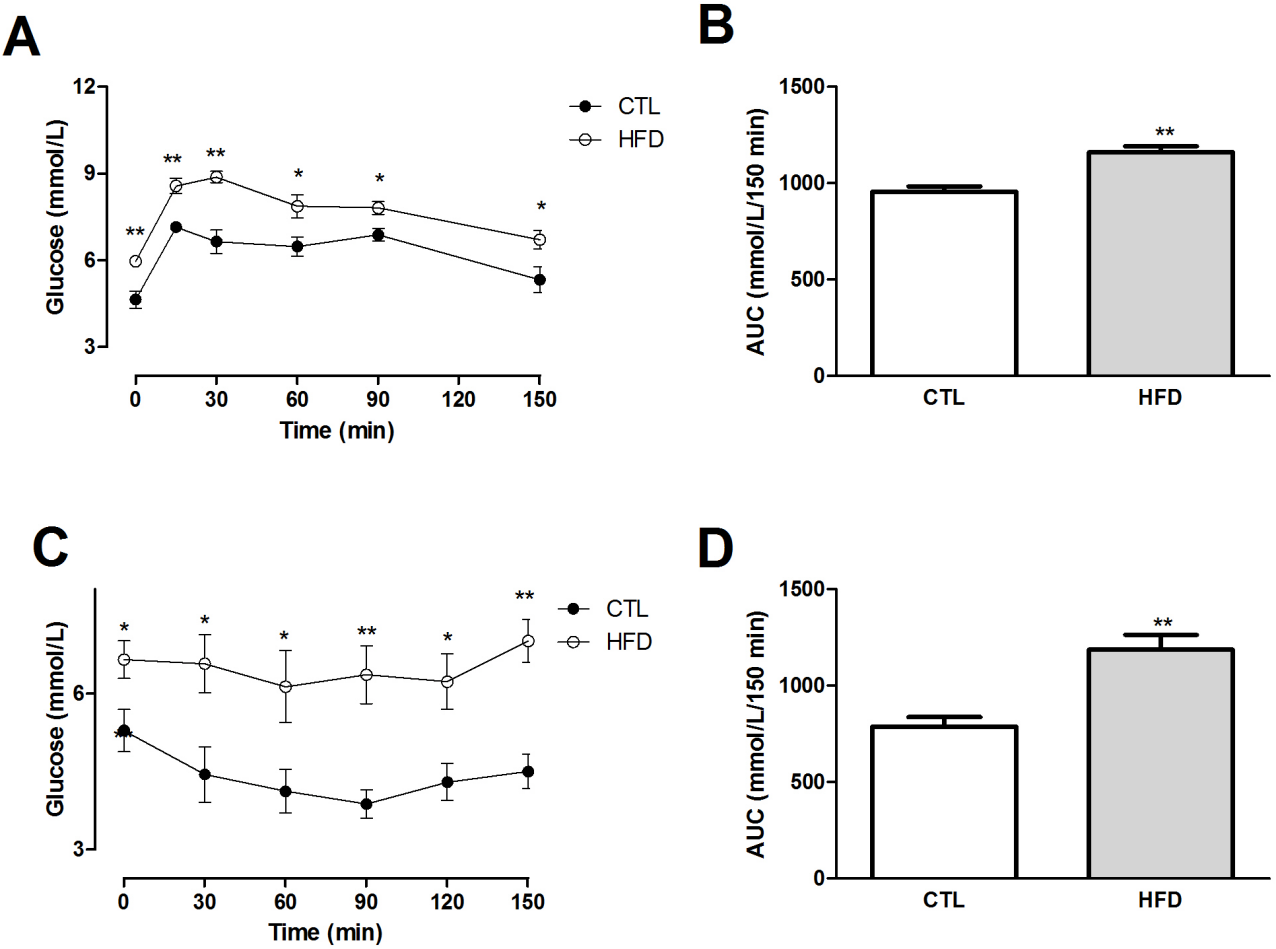
Plasma glucose levels during the oral glucose tolerance test and intraperitoneal insulin tolerance test. Oral glucose tolerance test (OGTT; A and B) (2 g/kg) or intraperitoneal insulin tolerance test (IpITT; C and D) (1U/kg) following overnight (OGTT) or 6 h (IpITT) of fasting in Wistar rats fed a standard chow (CTL) (•) or high-fat (◦) diet (HFD) for 20 weeks. Area under the curve (AUC) of glucose (B) and insulin (D) were calculated using the trapezoidal rule. Results are means ± SEM (n = 8–10). *P < .05 versus control; **P < .01 versus control.

**Fig 2:**
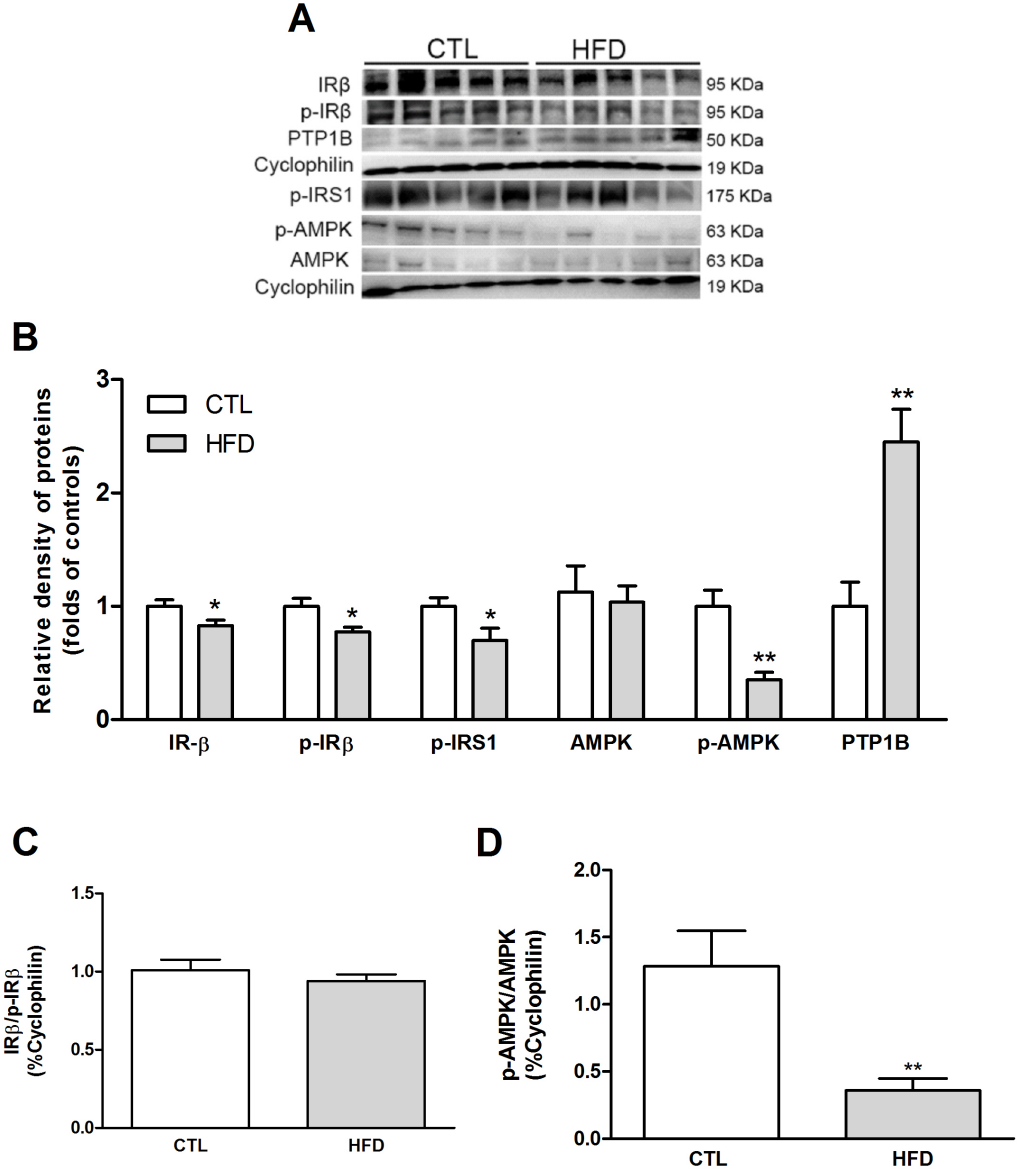
Insulin signalling components in the liver of NAFLD rat model. Expression of insulin signalling components in the liver of Wistar rats fed a standard chow (CTL) or high-fat diet (HFD) for 20 weeks was assessed by Western blot (A). The histogram represents means ± SEM of the densitometric scans for the protein bands of insulin pathway components, normalised by cyclophilin (B) and the ratio IRβ/p-IRβ (C) and p-AMPK/AMPK (D). *P < .05 versus control; **P < .01 versus control.

High-fat diet induced impairments in glucose metabolism, as evidenced by higher fasting blood glucose levels (Table 1), higher peak glucose after challenge and delayed return to baseline (Fig 1A and 1B). Also, HFD rats had a blunted response to insulin, indicative of insulin resistance as assessed by intraperitoneal insulin tolerance test (IpITT) (Fig 1C and 1D). High-fat diet induced impairments in insulin pathway as evidenced by a decrease in IRβ, *p*-IRβ and *p*-IRS1 protein expression in HFD-fed rats (Fig 2A and 2B). Total AMPK protein expression was not altered, while phosphorylated AMPK was decreased by 65% in HFD group (Fig 2A and 2B). The ratio p-IRβ/IRβ was not altered, while the ratio *p*-AMPK/AMPK was significantly decreased by 72% (Fig 2C and 2D, respectively). PTP1B protein expression was decreased by 145% after 20 weeks of HFD feeding (Fig 2A and 2B).

### Liver micro and macroscopic alterations after 20 weeks of HFD-feeding

**Fig 3:**
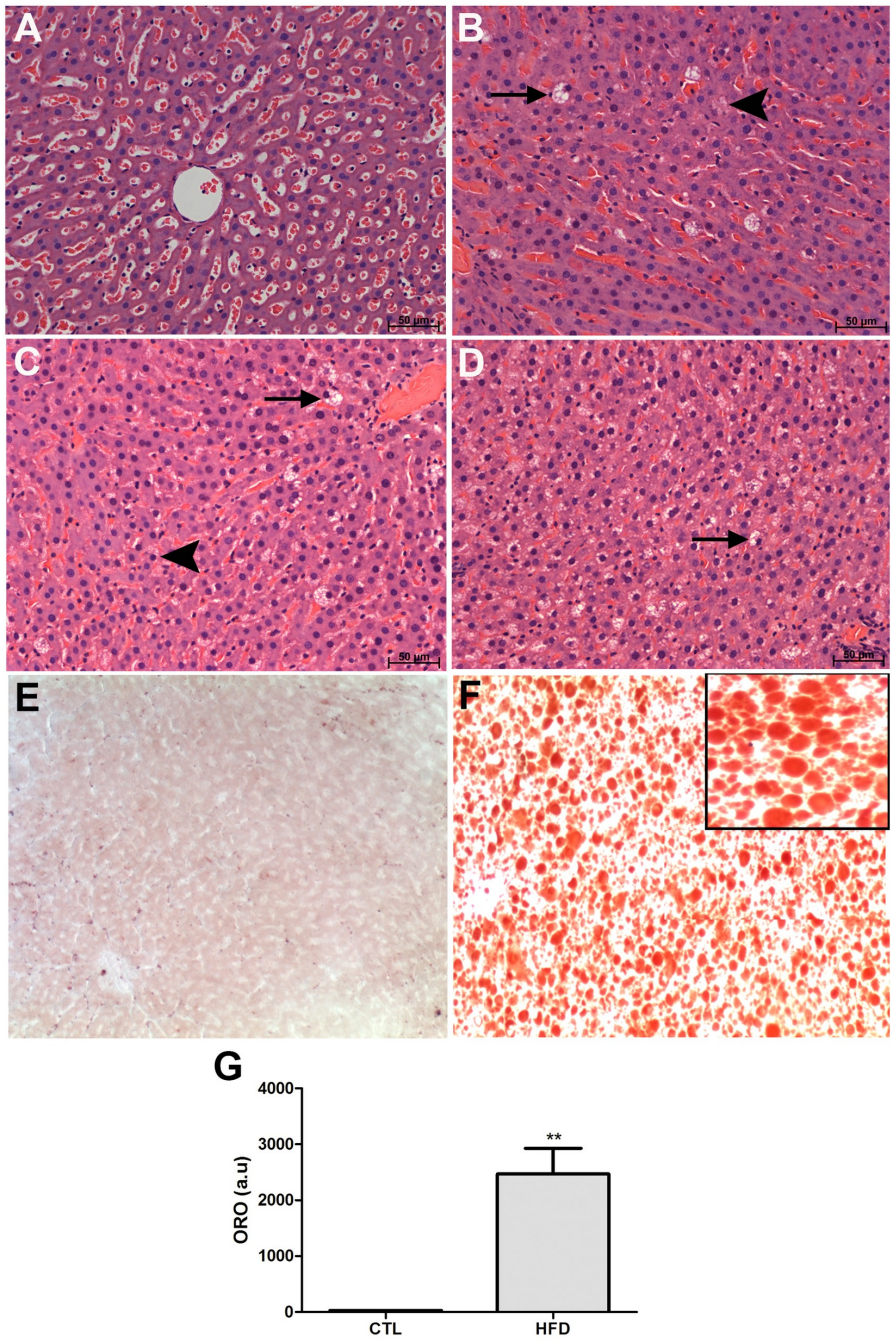
Evaluation of steatosis, inflammation and lipid deposition in the liver of HFD-fed rats. Representative images of livers from the control (A) and high fat diet (B, C, D) groups stained with Hematoxilin and Eosin or Oil-Red O staining. A. Control showing no histopathologic alterations. B. Macrovesicles in individual cells, suggesting macroesteatosis, surrounded by some microsteatotic cells. C. Moderate level of steatosis with several microsteatotic cells. D. Severe hepatic steatosis: almost all cells contain microvesicles and some contain macrovesicles. No fibrotic or inflammatory alterations were observed. Arrows indicate macroesteatotic cells and arrowheads microesteatotic cells. Neutral lipid accumulation in liver from control (E) and high fat diet (G) groups. Quantification of oil-red stained sections (G). Magnification is ×20. Insets from the images are magnified five times in order to highlight the steatosis and lipid-staining morphology. a.u., arbitrary units. **P < .01, compared with control rats.

Liver histology at 20 weeks confirmed the presence of steatosis, while no established necro-inflammatory lesions was evident (Fig 3A–3D). Histological analyses showed that 22.5% of HFD-fed rats presented severe steatosis, 22.5% mild and 55% moderate steatosis (data not shown). Lipids deposition in the liver significantly increased in HFD-fed rats (Fig 3E–3G).

**Fig 4:**
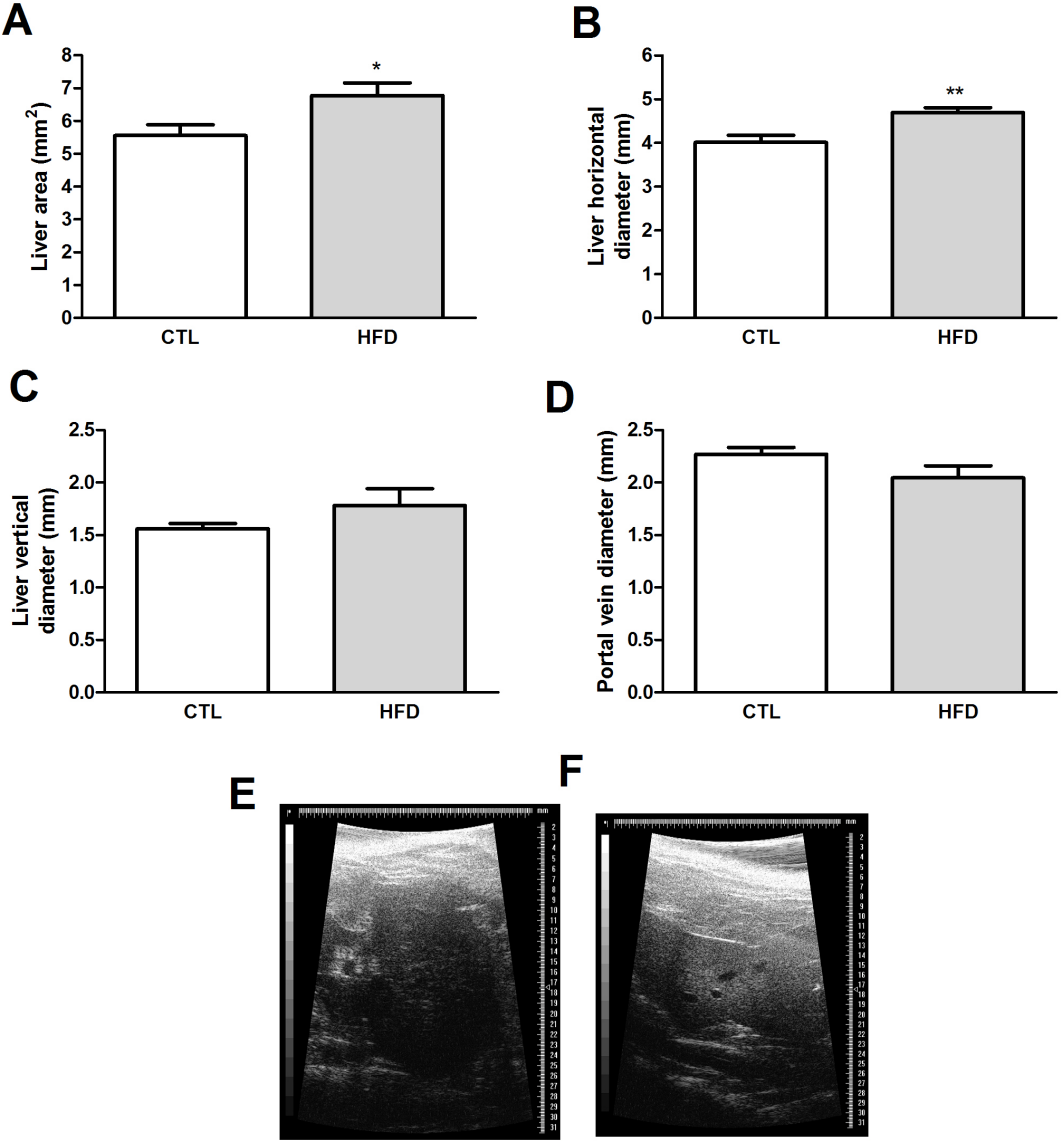
Ultrasonographic analyses of steatotic livers. Liver area (A), liver horizontal (B) and vertical diameter (C), portal vein diameter (D), liver ultrasonographic representative images of control (CTL)(E) and high-fed diet (HFD)-fed rats (F). *P < .05 versus control; **P < .01 versus control.

Liver ultrasound analysis revealed increased liver area (Fig 4A), horizontal diameter (Fig 4B) and parenchymal echogenicity (Fig 4E and 4F) in HFD-fed rats compared to controls. There were no changes in liver vertical diameter or portal vein calibre in both groups (Fig 4C and 4D, respectively).

### Liver microcirculatory dysfunction induced by HFD-feeding

**Fig 5:**
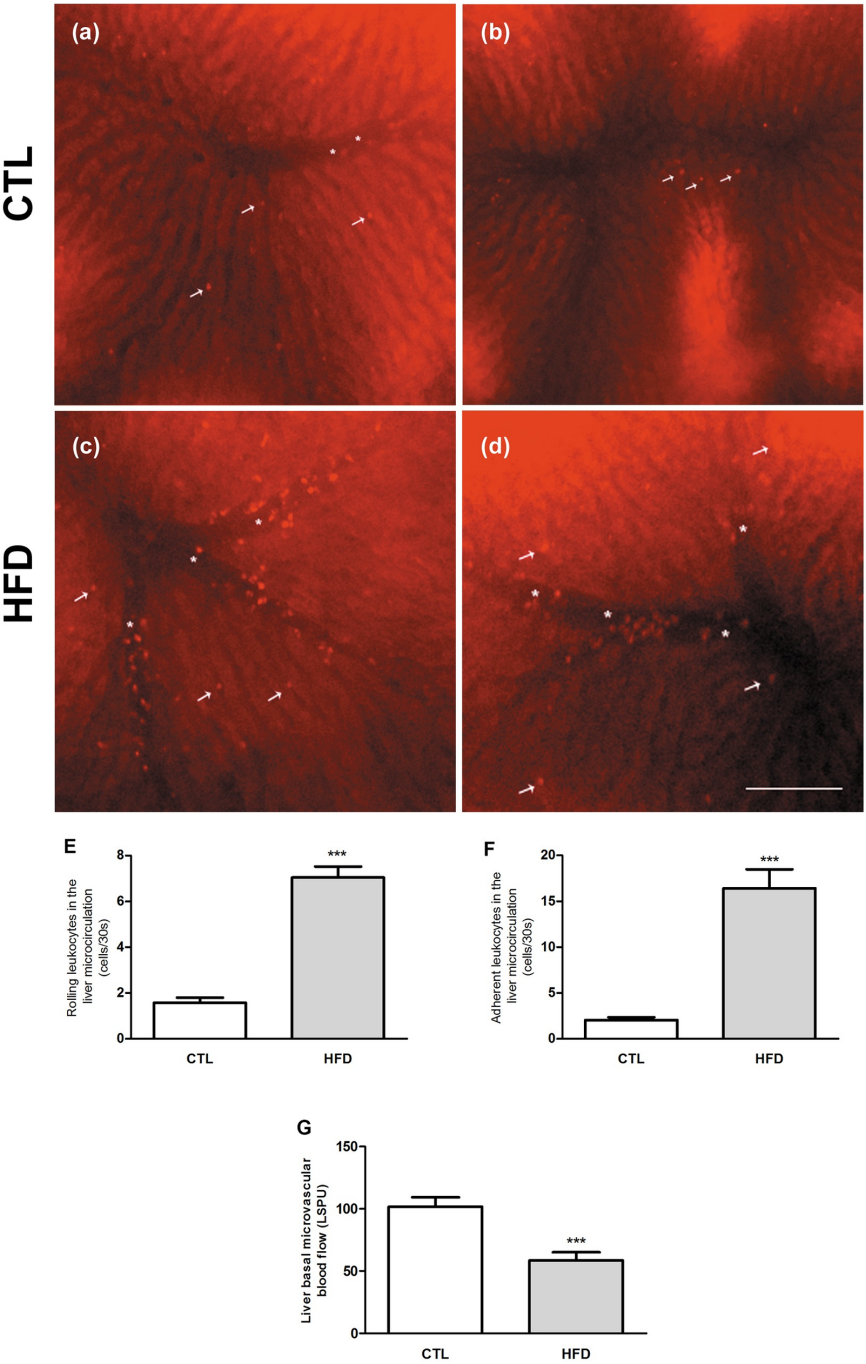
Hepatic microcirculation alterations in NAFLD. Representative image of the liver microcirculation of CTL (A and B) and HFD-fed rats (C and D) assessed by intravital microscopy; off-line quantification of rolling (E) and adhesion (F) of leukocytes in the sinusoids and post-sinusoids venules. Liver microvascular blood flow evaluated by laser speckle contras imaging (LSCI) is represented in G. Values are presented as the mean (± SEM). ***P < .001 versus control. Arrows indicate rolling/adherent leukocytes on sinusoids and asterisks rolling/adherent leukocytes on post-sinusoids venules.

Regarding the microcirculatory parameters, HFD causes a five- and eight-fold increase in leukocyte rolling and adhesion on hepatic microcirculation, respectively (Fig 5A–5F). In addition, LSCI analyses showed a 42% decrease in liver microvascular blood flow in HFD-fed rats compared to control diet-fed rats (Fig 5G).

### Oxidative stress and inflammatory pathways activation in liver of HFD-fed rats

**Fig 6:**
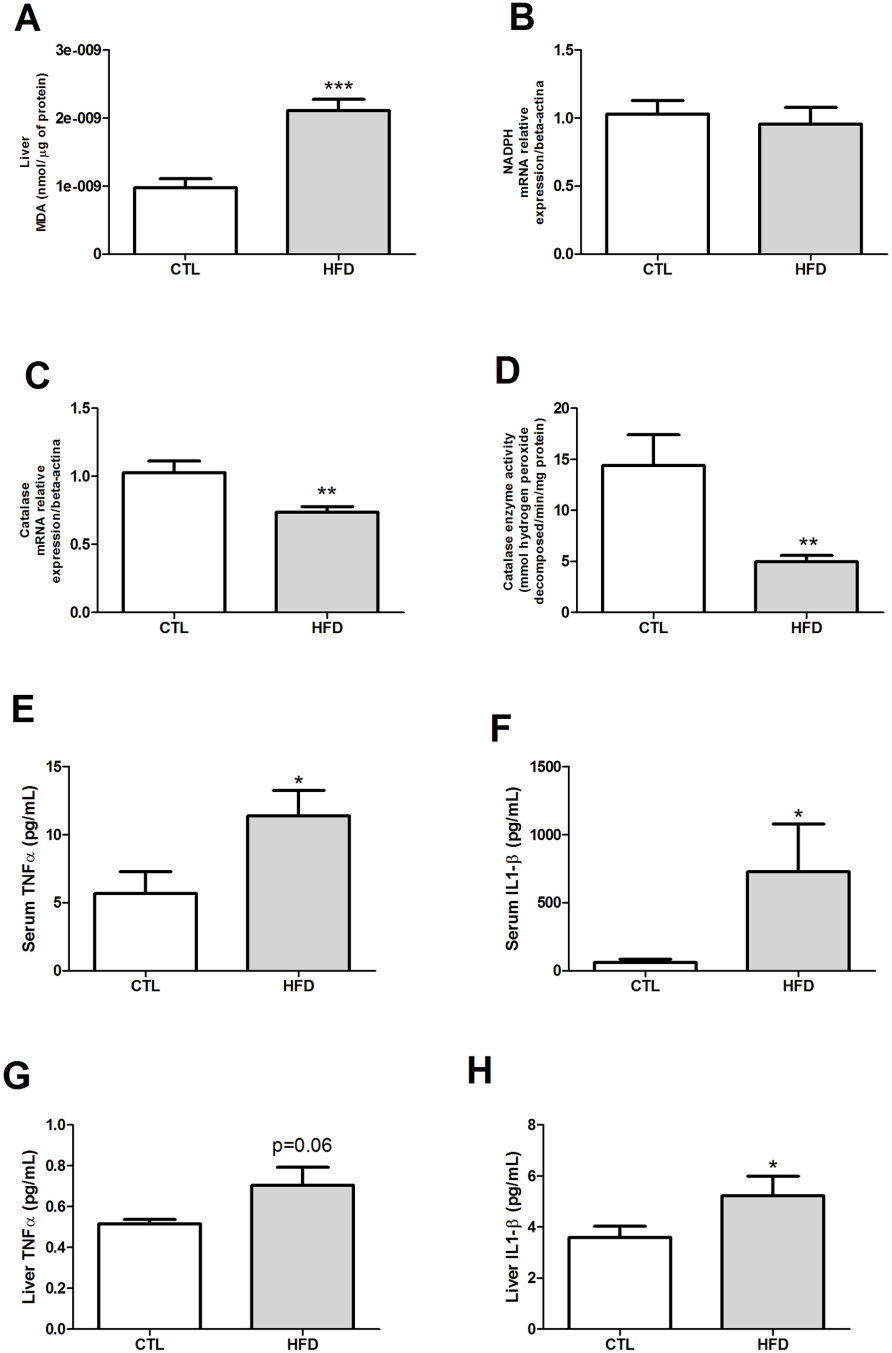
Oxidative stress and inflammatory parameters in the liver of control (CTL) and HFD-fed rats (HFD). Levels of malondialdehyde (MDA) indicating lipid peroxidation assessed by thiobarbituric acid reactive species (TBARs)(A); Real-time PCR analyses of mRNA transcript levels of genes coding for pro-oxidant gene NADPH oxidase (B) and anti-oxidant enzymes catalase (C). Catalase enzyme activity in the liver of control (CTL) and HFD-fed rats for 20 weeks (D). Serum and hepatic levels of TNF-α (E and G, respectively) and IL-1β (F and H, respectively) in control (CTL) and HFD-fed rats for 20 weeks assessed by ELISA quantification. *P < .05 versus control. **P < .01 versus control, ***P < .001 versus control. For this analysis at least six animals of each group were analysed and three independent experiments performed.

**Fig 7:**
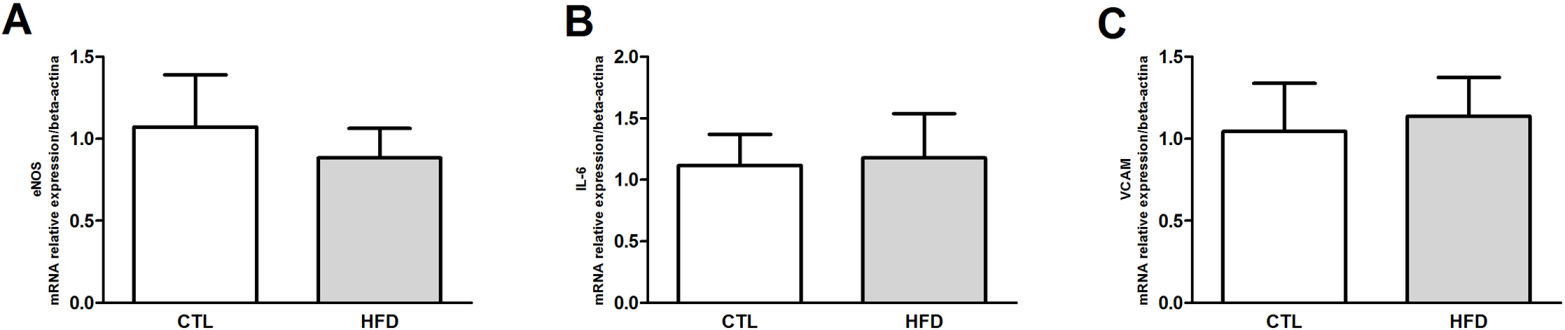
Gene expression of eNOS, IL-6 and VCAM after 20 weeks of HFD feeding. Real-time PCR analyses of mRNA transcript levels of genes coding for eNOS (A), IL-6 (B) and VCAM (C) in the liver of control (CTL) and HFD-fed rats for 20 weeks.

Total liver lipid peroxides (TBARs) were significantly elevated after 20 weeks of HFD compared with control diet-fed rats (Fig 6A), indicating higher levels of oxidative stress. NADPH gene expression was not altered in the liver of HFD-fed rats (Fig 6B), while catalase gene expression (Fig 6C) and activity (Fig 6D) were reduced in the liver by 30% and 65%, respectively. To determine whether inflammatory mediators were altered in the HFD group, the serum and liver concentrations of TNF-α and IL-1β were measured. In the HFD group the serum (Fig 6E and 6F) and liver (Fig 6G and 6H) concentrations of both cytokines were significantly increased compared to control group. Gene expression of IL-6, eNOs and VCAM were not altered in the liver of 20 weeks HFD-fed rats compared to control group (Fig 7A, 7B and 7C, respectively).

### Increased AGE-RAGE pathway activation in NAFLD

**Fig 8:**
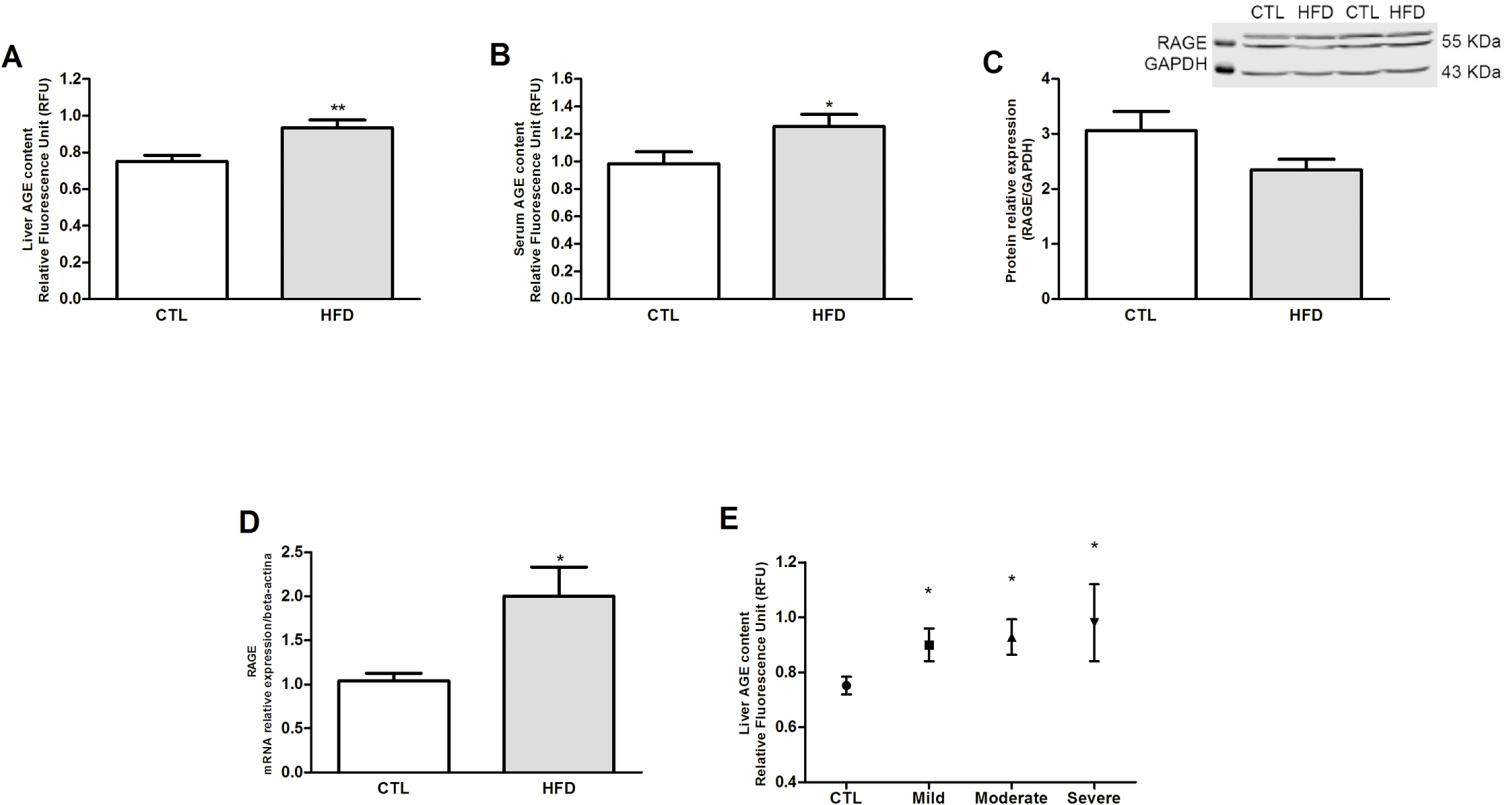
Participation of advanced glycation end product (AGE) and the receptor for advanced glycation end products (RAGE) in NAFLD. AGE deposition in the liver (A) and serum of control and HFD-fed rats (HFD)(B). RAGE protein (C) and mRNA (D) levels where compared between the groups. Liver AGE content *versus* severity of steatosis is shown in E. *P < .05 versus control; **P < .01 versus control.

At 20 weeks, there was a significant increase in AGE content in the liver and serum of HFD-fed rats compared to controls (Fig 8A and 8B, respectively), while RAGE protein expression was not altered in the liver (Fig 8C). On the other hand, RAGE gene expression was significantly increased in HFD-fed group (Fig 8D). There was no correlation between severity of steatosis and hepatic AGE content (Fig 8E).

## Discussion

The present study showed that rats subjected to twenty weeks of HFD feeding developed glucose intolerance, increased body fat deposition, and hypertension, in accordance with our previous results [26]. In addition, the HFD-fed rats presented rarefaction of cardiac and skeletal muscle microcirculation [26]. We presently investigated the liver alterations induced by HFD-feeding. We clearly show that livers of HFD-fed animals have features of NAFLD, and we propose this as an animal model to study the pathophysiology of NAFLD. The rats presented a cluster of liver alterations including steatosis (micro- and macrovesicular), increased triglycerides and cholesterol deposition, activation of oxidative stress and inflammation and an increase in liver area and echogenicity. Moreover, liver of HFD-fed rats presented an increase in AGE deposition and microcirculatory disturbances, which could play a critical role in NAFLD progression. At molecular level, insulin signalling impairment and decreased activation of AMPK are consistent with hepatic steatosis, inflammation and increase in ROS production [37,38]. Since AMPK signalling reduces *de novo* lipogenesis and stimulates fat oxidation [37,39], the suppression of the enzyme activity may potentially contribute to the pathogenesis of NAFLD. This is reinforced by studies showing that drugs stimulating AMPK activity ameliorate hepatic steatosis [37,40], evidencing that AMPK is a therapeutic target for NAFLD. However, previous work of Ouadda et al. (2009) were unable to show changes in the ratio of p-AMPK/total AMPK in the liver of obese *Psammomys obesus gerbils*, which develop either insulin resistant and diabetes [41]. Instead they found an increase in AMPKα1 mRNA and decrease in AMPKα2 mRNA levels which was correlated to the transition from insulin resistance to type 2 diabetes development. Here we did not evaluate specifically each isoform [41].

Microcirculation injury in the liver has been observed in alcoholic steatohepatitis (ASH), contributing to the development of portal hypertension [42,43,44]; however fewer studies addressed the hepatic microcirculation status in NAFLD. McCuskey et al. (2004), showed altered microcirculation in the liver of mice with NASH induced by methionine- and choline-deficient (MCD) diet, suggesting that factors other than ethanol might be responsible for microcirculation injury in the liver [20]. However the relevance of the MCD model to human disease is limited since the metabolic profile of MCD fed animals does not resemble human NAFLD, while rodent models based on long term HFD are associated with many of the metabolic features of human NAFLD [45]. Our study and that from Pasarín *et al* (2012) demonstrated substantial microvascular changes in early NAFLD, when solely simple steatosis is observed [46]. However, the microvascular abnormalities presently found were observed by *in vivo* microscopy and includes: a substantial decrease in blood flow through the hepatic sinusoids and a pronounced increase in rolling and adhesion of leukocytes in sinusoids and post-sinusoids venules, while the study from Pasarín et al (2012) showed impaired vasodilatory response of the liver vascular bed to acetylcholine in isolated and perfused liver of rats subjected to a Cafeteria diet [46]. *In vivo* approaches such as IVM and LSCI approaches can fully recapitulate the organ and systemic multicellular architecture, chemokine gradients, blood perfusion, and physicochemical properties in live animals significantly contributing to the understanding of pathophysiology in the context of living systems, in real time and in physiological conditions [47].

The factors that are reported to contribute to the alteration of blood flow in the liver could be many, including (i) enlargement of parenchymal cells due to lipid accumulation; (ii) secretion of vasoactive intermediates (such as nitric oxide, NO) by Kupffer cells; (iii) increase in reactive oxygen species (ROS) production; (iv) activation and contraction of the perisinusoidal stellate cells as well as (v) proinflammatory and profibrogenic cytokines; (vi) deposition of extracellular matrix proteins in the space of Disse and (vii) structural and functional changes in liver sinusoidal endothelial cells (LSEC)[7,43]. We did not observe suppression of eNOs expression. On the other hand, we found an increase in rolling and adhesion of leukocytes to the sinusoids, enlargement of parenchymal cells, increase in oxidative stress markers, and increase inflammatory mediators TNF-α and IL-1β, which can explain at least in part the changes in blood flow found in liver microcirculation. Further experiments in order to evaluate NO bioavailability and additional inflammatory and oxidative stress markers in the liver are needed to fully understand the mechanisms by which microcirculatory injury are generated in NAFLD. Irrespective of the precise mechanism of blood flow change in sinusoids it may exacerbate the hepatic injury by depriving the centrilobular regions of oxygen and impair the exchange of nutrients between hepatocytes and the vasculature as previously reported in obese Zucker rats [48].

AGEs and/or RAGE have been shown to be elevated in patients and experimental models of liver diseases [14,49,50,51,52]. Accordingly, we showed an increase in liver AGE content in HFD-fed rats. We did not find a correlation of liver AGE levels with the steatosis grade. Similarly, Hyogo et al., 2007 showed that AGE levels were significantly elevated in NASH patients compared to those with simple steatosis, however the AGE levels were no different with the steatosis grade of those patients [13]. Leung et al (2016) showed similar results using high-AGE diet induced experimental NAFLD [16]. According to the multiple hit hypothesis of NASH progression several parallel factors synergistically contribute to disease progression from benign steatosis to steatohepatitis [53,54]. Leung et al., 2014 showed that NAFLD experimental model induced by MCD-deficient diet, showed increased hepatic AGE content and an AGE/RAGE dependent increase in ROS production by Kupffer cells [11]. High dietary AGEs have been shown to increase hepatic AGE content, exacerbate liver injury and increases in the levels of pro-inflammatory cytokines via AGE/RAGE dependent activation of HSC and subsequently increase in oxidative stress and fibrosis [10,16]. AGEs-driven increases in oxidative stress and tissue inflammation suggests that AGEs play an important role in the “multiple hit hypothesis” and could participate in microcirculatory injury herein observed in liver of HFD-fed rats [49]. This is reflected in our finding that liver of HFD-fed rats presented increased MDA content, suggesting increases in lipid peroxidation, decreased anti-oxidant capacity and increased inflammatory markers TNF-α and IL-1β. The increase in oxidative stress and inflammatory markers observed in our HFD-fed rats could be due and/or amplified by the increase in AGE deposition in the liver, and thus contribute to the liver microcirculation impairment.

Importantly, since Kupffer cells and LSEC are the major cellular sites of AGE uptake and clearance [24], liver dysfunction, including microcirculatory disturbances, will impair AGE metabolism, leading to further increase in plasma concentration of AGEs in patients with liver diseases [13,55]. Whether the microcirculatory disturbances are reversible or irreversible is unclear and should be addressed since there are many concerns about the use of steatotic liver for transplantation [56]. The microcirculation of steatotic liver is more sensitive to the ischemia-reperfusion (IR) injury than normal liver [56], reinforcing the critical role of microcirculatory alterations in NAFLD.

In summary, herein we showed that the increase in liver AGE levels and microcirculatory disturbances are involved in the liver injury induced by HFD feeding and that AGE-RAGE axis, oxidative stress and inflammation could have a role in microcirculatory alterations in NAFLD. Further studies are needed to precisely determine the pathogenic roles for the AGE-RAGE axis in liver microcirculation in NAFLD.
